# Correction: Population dynamics of western gorillas at Mbeli Bai

**DOI:** 10.1371/journal.pone.0279608

**Published:** 2022-12-19

**Authors:** The PLOS ONE Staff

There are errors in the author affiliations. The correct affiliations are as follows:

Andrew M. Robbins^1^, Marie L. Manguette^1,2^, Thomas Breuer^2,3^, Milou Groenenberg^2^, Richard J. Parnell^4^, Claudia Stephan^5,6,7^, Emma J. Stokes^4^, Martha M. Robbins^1^

**1** Department of Primatology, Max Planck Institute for Evolutionary Anthropology, Deutscher Platz 6, 04103 Leipzig, Germany, **2** Mbeli Bai Study, Wildlife Conservation Society Congo Program, B.P. 14537, Brazzaville, Congo, **3** World Wide Fund for Nature, Reinhardtstrasse 18, 10117 Berlin, Germany, **4** Wildlife Conservation Society, Global Conservation Program, 2300 Southern Boulevard, Bronx, NY, 10460, USA, **5** Wildlife Conservation Society–Congo Program, Republic of Congo, **6** Nouabalé-Ndoki Foundation, Republic of Congo, **7** Division of Developmental Biology, Friedrich-Alexander University Erlangen, Germany. The publisher apologizes for the errors.
