# Supplementary material for: Population dynamics of western gorillas at Mbeli Bai
Source: PLoS One. 2022 Oct 19;17(10):e0275635. doi: 10.1371/journal.pone.0275635 (PMC9581538; doi:10.1371/journal.pone.0275635)
Supplement: S1 File — (DOCX) [file pone.0275635.s001.docx]

Supporting Information for the population dynamics of western gorillas

Section S1. Additional growth rate calculations

Section S2. Influx and outflow

Section S3. Density dependence

Section S4. Social structure and age structure

Section S5. Frequency of gorilla visits to the bai

Section S6. Methods for the Supporting Information

Section S7. Tables for the Supporting Information

Section S8. Figures for the Supporting Information

Section S9. References for the Supporting Information

**Section S1. Additional growth rate calculations**

This section presents additional growth rate estimates for the Mbeli western gorillas, as well as comparisons with the mountain gorillas at Karisoke and Bwindi. The estimates are based on Leslie matrix models that use previously published birth rates and survivorship curves (See Figure S1.1 and the Methods in Section S6).

The Leslie matrix calculations predicted a growth rate of 0.0% for the Mbeli western gorillas, which fits within the 95% confidence limits of -0.7% to 2.6% that we estimated using time-series analyses (see the main text). The models predicted a growth rate of 3.0% for the Karisoke mountain gorillas, which is slightly less than previous estimates of 3.1% to 4.1% for a broader population of all habituated gorillas in the Virungas [1]. The Leslie matrix calculations predicted a growth rate of 1.7% for the Bwindi mountain gorillas, which is less than the previously published range of 2.5 to 4.4% that was based on time-series analyses [2]. Discrepancies between the estimates within each population could reflect spatial and/or temporal differences in scope of the datasets, as well as the differences in methodology. The time-series analyses quantify how a population size has changed in the past, whereas the Leslie matrix calculations predict what the growth rate would be if a population maintained the same survivorship curves and birth rates for long enough to achieve a stable age structure [1, 3].

The estimated growth rates suggest that Karisoke has been farthest from its carrying capacity, followed by Bwindi, and then Mbeli is at or near its carrying capacity. As a population density increases towards its carrying capacity, changes in demographic rates are predicted to follow a progression, starting with higher mortality rates for immatures, followed by a later age of first reproduction, then lower reproductive rates (e.g., longer interbirth intervals), and finally higher mortality rates for adults [4]. If differences in population density (relative to carrying capacity) are the primary reason for differences in demographic rates among the gorilla populations, then we might expect those rates to follow the same progression from Karisoke to Bwindi to Mbeli. For example, we might expect the first 1-2 parameters to be the main difference between Karisoke and Bwindi, and then additional parameters could differ with Mbeli.

Rather than following expected progression, however, those four parameters generally seem to shift together among the gorilla populations (Figure S1.2). Three of the four parameters are higher for Bwindi than Karisoke, and all four are higher for Mbeli than Bwindi. The only exception is the age of first reproduction, which is essentially identical for Karisoke and Bwindi, even though it was expected to be the second parameter to start changing. Hypothetically, the predicted progression in demographic parameters might be more apparent when examining continual changes within a population, rather than comparing differences among populations. As shown in the main text, however, we found no significant changes in demographic parameters at Mbeli, even as the population doubled in size.

Hypothetically, we might also expect to find greater differences in the parameters that were predicted to start changing first. In other words, the order of the data points for Mbeli (from top to bottom) would match the order that the parameters are listed in the legend of Figure S1.2. Those expectations were matched by some results for infant mortality, which showed the greatest difference between Mbeli and Karisoke, and is expected to start changing soonest. Similarly, the smallest difference between Mbeli and Karisoke occurred with adult mortality, which is expected to start changing last. For the age of first reproduction and reproductive rates, however, the relative differences between Mbeli and Karisoke do not match the order in which they were expected to start changing. In addition, none of the relative differences between Bwindi and Karisoke match the order in which they were expected to start changing. A previous comparison between Bwindi and Karisoke had found differences in interbirth intervals, but not infant mortality, which was considered inconsistent with the predicted patterns for differences in population density (relative to carrying capacity). This more comprehensive comparison among three populations has shown additional differences in demographic rates that again may not fully reflect differences in relative carrying capacity.

In addition to differences in population density (relative to carrying capacity), genetic variations can also contribute to demographic differences among populations. Among the three populations in this study, genetic variations are expected to be greatest between mountain gorillas versus western gorillas, which are classified as separate species. The mountain gorillas at Bwindi and Karisoke are considered to be the same subspecies, but some genetic differences could exist because the two populations have been isolated from each other since they were discovered.

Genetic variations among populations have been expected to create a continuum of life history patterns, whereby species with a “fast” life history will have higher birth rates and higher mortality rates than species with a “slow” life history [5]. This continuum is represented by the diagonal line in Figure S1.3, where the birth rate equals the mortality rate, as expected when the growth rate is zero (e.g., the “M” represents the Mbeli population). Growth rates will be positive for populations above the diagonal (e.g., the “K” for Karisoke and the “B” for Bwindi), and negative for populations below the diagonal.

Because the three closely related gorilla populations have different growth rates, it is not straightforward to predict where their life history traits would fall along a continuum at the same growth rate. Hypothetically, for example, the Karisoke population could approach its carrying capacity by exclusively increasing its mortality rate, as indicated by the horizontal dashed line toward the point labeled “Km” in Figure S1.3a. Alternatively, Karisoke could approach its carrying capacity by exclusively decreasing its birth rate, as indicated by the vertical dashed line toward the point labeled “Kb”. The Mbeli population falls well within the range between Km and Kb, so it is not obvious whether Karisoke would have a faster or slower life history than Mbeli at the same growth rate (i.e., at their respective carrying capacities). The Mbeli population would also fall within the corresponding range for Bwindi (red dashed lines toward Bm and Bb), which again falls within the range for Karisoke.

As explained above, populations are typically expected to approach their carrying capacity through a progression of demographic changes that involve both the birth rate and the mortality rates. (The age of first reproduction is incorporated into the birth rate that is shown in Figure S1.2 – see the Figure legend for details). If those birth rates and mortality rates changed in equal proportions, then the Karisoke population would approach its carrying capacity along the blue dotted line in Figure S1.3b, which ends close to the point for the Mbeli population. In this scenario, we could conclude the current differences in life history parameters between Karisoke and Mbeli are primarily due to their population density (relative to carrying capacity), rather than any genetic differences between species. We could reach a similar conclusion about differences between Bwindi and Mbeli, although Bwindi would seem to have a slightly slower life history. Such a conclusion would contrast with the prevailing suggestion that mountain gorillas have a faster life history than western gorillas. The prevailing perspective is based on the higher reproductive rates and earlier maturation for mountain gorillas, but those differences could mainly indicate that mountain gorillas are currently below their carrying capacity.

As we have shown, demographic parameters do not necessarily follow the expected progression as a population approaches its carrying capacity, and the changes may depend on factors such as nutritional sufficiency, extrinsic mortality risks, and socioecology [6]. For example, the growth rate of the Karisoke mountain gorillas has been reduced by male mating competition, as an increase in group density led to higher infanticide and silverback mortality [7]. An increase in infanticide could lead to higher birth rates, because females give birth sooner after an infant dies [8]. Thus, infanticide could create an even greater range of uncertainty along the fast-slow continuum (see the point labeled “Ki”in Figure S1.3b), along with a greater possibility that mountain gorillas have a faster life history than western gorillas. From this perspective, it becomes even harder to evaluate how genetic variations contribute to the current life history differences among the gorilla populations. We can be certain, however, that changes in life history parameters are needed to alter a growth rate, so deviations from carrying capacity must be contributing to those differences.

Ideally, differences in growth rates would be considered in other phylogenetic analyses of life history variations among species. Our results probably represent an extreme example, however, because we are comparing only three very closely related populations, including two that are considered the same subspecies. Phenotypic plasticity within populations should become less important when evaluating a broader range of species, such as comparing gorillas with pygmy mouse lemurs [9]. Phylogenetic analyses typically involve many more species too, so their results will be less sensitive to the growth rate of any particular population. If so, then differences in growth rates may merely contribute to the error term in most phylogenetic analyses, without creating any systematic bias in their conclusions.

**Section S2. Influx and outflow**

This section provides more details about the influx and outflow of gorillas between the study population and the surrounding area. As mentioned in the main text, those exchanges can be divided into four mechanisms:

1. Locational dispersal of groups
2. Locational dispersal of solitary males
3. Voluntary dispersal of subadult and adult females
4. Involuntary dispersal during group disintegrations

1) As shown in the main text, the influx of gorillas mainly involved immigration of individuals into existing study groups (social dispersal), but it also included the appearance of a few previously unknown groups (locational dispersal). Overall, 44 of the 512 gorillas in this study arrived via locational dispersal. Those gorillas were identified in 25 social units, which included 3 breeding groups, 8 non breeding groups, and 14 solitary males (Table S2.1). When they were identified, the immigrant groups already contained an additional 18 gorillas that had transferred from study groups. In 5 of the 11 groups, the only new gorilla was the silverback, so those cases could be considered locational dispersal of a solitary male who subsequently acquired a group in the area (rather than locational dispersal of an entire group). Two of the three breeding groups contained only one infant, with no juveniles or subadults, which suggests that they may have formed recently too. Thus, the locational dispersal could mainly reflect solitary males and new groups that are starting to establish their home ranges, rather than human disturbances that are forcing established groups to leave the adjacent areas. In contrast, two of the greatest shifts in home range among the habituated Virunga mountain gorillas occurred with groups that had been observed for over ten years, which were potentially associated with competition from other social units [10-12]. We found no evidence of study groups leaving the area. Strong home range fidelity has also been observed among groups of western gorillas in the Loango National Park in Gabon [13, 14].

2) It is difficult to determine whether the locational dispersal of solitary males had a net influx or outflow, because their unexplained disappearances could have been either deaths or locational dispersal out of the study (outflow). Similarly, the unexplained disappearances of some subordinate males could involve locational dispersal, if they emigrated from their group, and then disappeared before they were observed as solitary. To further evaluate the potential cases of locational dispersal, we compared the unexplained disappearances of subordinate and solitary males against their alternative fates (Figure S2.1). The eight subordinate blackbacks and silverbacks that disappeared from study groups had an average age of 14.8 ± 1.9 SD years, which is significantly younger than 16.5 ± 1.4 years for the 29 cases of known emigration (t = 2.4, df = 9.3, p=0.041). The earlier ages of subordinate disappearances suggests that some of them could have been deaths, especially before the youngest known emigration at age 12.6.

Following the known emigrations, seven solitary males disappeared at an average age of 16.6 ± 1.2 years, which is significantly less than the average age when ten other emigrants formed a successful breeding group (20.1 ± 2.2 years, t = 4.2, df = 14.3, p < 0.001). Those age estimates should be considered tentative, however, because many males have not yet disappeared, emigrated, and/or formed a group (Figure S2.2). The length of the solitary phase was not significantly correlated with the age of emigration, so later emigrations could lead to later age estimates for solitary males to disappear or form groups (R^2^ = 0.07, F_8,1_ = 0.59, p = 0.46). If all of the unexplained disappearances of blackbacks and young silverbacks were due to dispersal, then the study population would have an influx: outflow ratio of 27:36 for those males (i.e., a net outflow). If more than nine of the 37 unexplained disappearances were due to death (23%), then the study population would have a net influx.

3) As shown in the main text, the Mbeli population had a net influx of subadult females during the study, but the results were less conclusive for adult females. To further evaluate whether adult females had a net influx or outflow, we compared their cases of known dispersal and unexplained disappearances with the subadults females. According to the Leslie matrix calculations, adult females had a mortality rate of 0.036 deaths per female year, which would mean that approximately 48 of their unexplained disappearances were deaths, and the other 25 were outflow (Table S2.2). If so, then 18% of the 140 transfers by adult females in study groups would be outflow, which is not significantly different from 14% of the 22 transfers by subadults (Fisher exact test, p = 0.77). The ratio of influx : outflow for adult females would be 62:25, which is not significantly different than 22:3 for subadult females (Fisher exact test, p = 0.11). Thus, the transfer patterns of adult females seem to resemble the subadults, and both age classes may have had a net influx during the study.

4) As mentioned in the main text, the study population had a net influx of juveniles and subadult males, even if all of the unexplained disappearances during group disintegrations were due to outflow. To further understand the potential for outflow during group disintegrations, we examined the fate of gorillas in each age/sex category (Table S2.3). The analyses include 112 subordinate gorillas that were in 18 groups when the dominant male died (Table S2.4). Only 53% of the infants permanently disappeared during disintegrations, which supports previous reports that infanticide does not always occur when the dominant male dies [8, 15]. Among juveniles and subadults, 23% of the males disappeared which is not significantly different from 36% for females (coefficient = 0.14, standard error = 0.17, t = 0.82, p = 0.40). A higher proportion of disappearances could have been expected for the males if they are not always accepted into breeding groups, because they might need to travel farther to find a tolerant destination [16]. When the destination of juveniles and subadults was known, however, the proportion of males that joined a breeding group was essentially identical to the females (71% for both sexes, coefficient = 0.004, standard error = 0.19, t = 0.018, p = 0.98). Among older gorillas in the group disintegrations, 9.1% of the blackbacks and young silverbacks disappeared, which is significantly less than 32% for the adult females (coefficient = 0.23, standard error = 0.11, t = 2.2, p = 0.029). None of the blackbacks and young silverbacks joined another breeding group, at least 27% of them became solitary, and the rest joined a nonbreeding group or solitary male. Following the death of a group’s silverback, no males became dominant and sired offspring in their previous group, even though they were as old as 17.7 years, which is beyond the age when males acquire groups in the Virungas [17, 18].

**Section S3. Density dependence**

As stated in the main text, we found no significant evidence of density dependence in female reproductive success as the population size doubled. To continue looking for evidence of density dependence, we estimated a separate value for the population growth rate in each year of the study, based on an approach that was previously used for mountain gorillas [7]. The calculations focus on the number of births and deaths, relative to the population size, so they should not be biased by influx and outflow (See the Methods in Section S6). According to those results, the population growth rate did not vary significantly among the years of the study (Figure S3.1). The results are consistent with the main text, which showed that the underlying demographic rates for births, deaths, and disappearances also did not vary significantly among the years of the study. The average value of the annual growth rates equaled 0.8%, which is again consistent with the results from the main text.

The apparent absence of density dependence may suggest that the population is not near its carrying capacity, but there are a few other hypothetical possibilities.

- One possibility is that the carrying capacity has been increasing due to improved habitat quality. The Ndoki-Likouala landscape seems to have a gradient in gorilla densities, which may be related to the distribution of terrestrial herbaceous vegetation [8, 19, 20]. We have no evidence that the vegetation distribution has shifted to improve the habitat quality at Mbeli.
- Another possibility is that the carrying capacity has been increasing due to reduced predation. Western gorillas have been attacked by leopards, which are present in the NNNP, but the impact on population dynamics is not known [21-23]. We have no evidence of reduced predation at Mbeli, and we would still expect to see density dependence in female fertility as the population increased.
- Our analyses assumed that the study area remained constant, so a third possibility is that the study groups actually doubled their collective ranging area and displaced the surrounding gorillas to maintain a constant density as the population increased. Again, we have no evidence of this possibility, which seems unlikely if some of the surrounding areas already have higher density than Mbeli.

The details for those hypothetical possibilities are far from conclusive, so they are merely presented to illustrate that insufficient information is currently available.

**Section S4. Social structure and age structure**

This section provides additional details about the population dynamics for social structure and age structure. Among the 55 groups that were observed during the study, 13 were present when the study began (24%), 11 appeared through locational dispersal (20%), 29 began when a solitary male formed a new group (53%), and two groups were created when another group fissioned (Table S4.1). Among the 55 study groups, 36% ended through disintegration, 13% ended through attrition, one group (2%) ended in the fission, and 49% were still present at the end of the study.

During the study, 58% of the groups were always observed as breeding groups, 9% were exclusively nonbreeding groups, and 33% were observed in both categories. Solitary males formed 22 breeding groups when they acquired adult females, and they formed seven nonbreeding groups when they were joined by other males and/or immature females. Nonbreeding groups became breeding groups when they acquired adult females, or when a subadult female reached adulthood (Table S4.2). There were 105 immigrations of adult and subadult females into social units via voluntary dispersal, along with 17 immigrations via involuntary dispersal, and 102 immigrations were not classified because they came from a nonstudy group (Table S4.3).

Among the adult and subadult female immigrations with a known origin, the relative frequency of voluntary versus involuntary immigration did not vary significantly among the three types of destinations: breeding groups, nonbreeding groups, and solitary males (GLMM: *Χ*^2^ = 1.7, df=2, p=0.44). After also including immigrations with an unknown origin, the immigration rate was 0.466 immigrations per group year for breeding groups, which is significantly higher than 0.189 for nonbreeding groups and 0.144 for solitary males (rate-based *Χ*^2^ = 11.2, df=2, p<0.001). Females typically transferred to a different silverback after an average of three births (Figure S4.1).

The age structure within a group often follows a predictable pattern over time, as solitary males acquire females, then sire infants, who mature into juveniles, subadults, and beyond [24]. Therefore, among groups that were observed since they were formed, the age of the group was highly correlated with the age of their oldest remaining offspring (r = 0.88, Figure S4.2). We used the correlation to estimate the age of 20 groups that had already formed before they were observed, and we included those estimates in some of the subsequent analyses (See the Methods in Section S6).

The 55 study groups were observed for a cumulative total of 454.1 group-years, which represents an average of 8.3 observation-years per group. The average length of observations does not fully reflect the actual lifespan of groups, however, because the entire duration was observed for only twelve groups (22%). Based on the groups whose formation was observed, a Kaplan Meier analyses indicates a median duration of 12 years, but that analysis could be considered tentative due to small sample sizes beyond age 12 (dots in Figure S4.3). When we include the estimated ages of groups that had already formed before they were observed, a life table analysis suggests a median duration of 12-15 years (red line in Figure S4.3). The 73 solitary males were observed for a cumulative total of 282.6 gorilla-years, which represents average of 3.9 observation-years per solitary male (Table S4.4). Again, the average length of observations does not reflect the actual length of the solitary phase, because only 29% of solitary males were observed from emigration to group formation, and because it is unknown when the solitary phase ends in death.

The average group size was 8.5 ± 3.9 SD for breeding groups, which is significantly higher than 5.0 ± 2.7 for nonbreeding groups (Figure S4.4). Based on a snapshot of the population on the first day of each year, 65% of the variance in breeding group size occurred among groups, with the remaining variance arising from temporal variations within each group. Group size was positively correlated with the elapsed time since a group began, and with the time until a group disintegrated (Figure S4.5). The probability that a group contained adult females was also positively correlated with the time since the group began (N = 2628 data points in 29 groups, Chi-sq = 482, p < 0.001), and for the time until the group disintegrated (N = 1774 data points in 19 groups, Chi-sq = 30.9, p < 0.001). Thus, groups were more likely to be breeding groups in the middle of their lifespan (versus when they started and ended).

**Section S5 – Frequency of gorilla visits to the bai**

To provide yet another perspective on the population dynamics of western gorillas at Mbeli, we examined temporal variations in the relative frequency of observed gorilla visits to the bai. This perspective allows us to account for any gorillas who visited the bai but were never sufficiently identified to be included in the demographic database. It would also include any visits when a gorilla in the database was not recognized (e.g., if observers did not get a good look at a gorilla who briefly entered the bai). This perspective does not allow us to control for influx and outflow between the study groups and the surrounding population, however, so it is more comparable to the rate of population increase, rather than the inherent growth rate.

For each year from 1996 to 2015, we defined the “relative visitation frequency” as the cumulative number of times that a gorilla was observed at the bai, divided by the number of observation-hours. Thus, this perspective also controls for temporal variations in observation effort, which becomes relevant because we are analyzing the number of observations. Each visit by a group was treated as a visit by each of its members that was observed.

When the analysis was limited to visits by identified gorillas in the demographic database, the relative visitation frequency was 0.64 gorilla visits per observation-hour in 1996, and it increased to 1.21 visits per observation‑hour in 2015 (Figure S5.1a). After log transformation, the slope of a regression line indicates that the relative visitation frequency increased by an average of 2.57% per year. The slope is similar to a 2.75% rate of increase in the size of the study population during the same years. (In the main text, Figure 1 shows those results over a broader time period). Thus, the number of gorillas in the study population seemed to increase at a rate that is consistent with their collective number of visits per year. In other words, we do not see evidence of any major changes in the visitation frequency per gorilla.

When we included visits by unidentified gorillas, the slope of the regression line indicated that the relative visitation frequency increased by an average of 2.52% per year (Figure S5.1b). Thus, the unidentified gorillas did not have a meaningful influence on our estimate of the increase in the relative visitation frequency. Those results suggest that the unidentified gorillas also would not have a meaningful effect on our estimate of the rate of population increase, even if they could be incorporated into the demographic database. This conclusion is not surprising because unidentified gorillas accounted for only 1.4% of the observed gorilla visits to the bai.

**Section S6. Methods for the Supporting Information**

***Methods for Section S1***

The additional growth rate estimates for the Mbeli western gorillas are based on Leslie matrix models, using birth flow calculations because gorillas are not seasonal breeders [1, 3]. The Leslie matrix models use previously published survivorship curves that were estimated through Bayesian analyses which considered the uncertainty in age estimates and the fate of unexplained disappearances (Figure S1.1). The models also use previously published birth rates: 0.255 births per adult female year for the Karisoke, versus 0.211 for Bwindi, and also 0.211 for Mbeli [1, 2, 25]. Those birth rates were applied throughout the ages when females are considered adults, which begins at age eight for mountain gorillas and age ten for western gorillas (ibid). All of the growth rate estimates assumed a 50:50 birth sex ratio [26].

***Methods for Section S2***

To compare the ages of unexplained disappearances versus known dispersal, we ran a t-test with one data point for each blackback and subordinate silverback that left a study group. To compare the ages of unexplained disappearances versus group formation, we ran a t-test with one data point for each solitary male that emigrated from a study group. The analysis excluded males that were still solitary when observations ended. Among the solitary males that formed a group, we ran a linear regression to compare the length of the solitary phase against their age of emigration.

We ran Fisher exact tests to compare the transfers by subadult females versus adult females. The first test compared the proportion of transfers among study groups, versus the transfers from a study group to the surrounding population (outflow). The second test compared the outflow versus the influx (i.e., the transfers from the surrounding population into a study group). In both cases the estimated outflow was based on the mortality rate from Leslie matrix calculations, the number of female-years observed, and the number of unexplained disappearances (Table S2.2).

We ran a Generalized Linear Mixed Model (GLMM) to compare the probability of disappearances for blackbacks and young silverbacks versus adult females during a group disintegration. The model included one data point for each of those gorillas during each group disintegration. The response variable equaled “1” if the gorilla disappeared, and “0” if it did not. The predictor variable was the sex of the gorilla. The model included a random effect variable for the identity of the group that disintegrated. We ran a similar GLMM to compare the probability of disappearances versus sex for juveniles and subadults during a group disintegration. When the gorilla did not disappear, we ran a third GLMM to compare the probability of joining a breeding group for juvenile and subadult males versus females. The response variable equaled “1” if the gorilla joined a breeding group, and “0” if it did not.

***Methods for Section S3***

To look for density dependence in the population growth rate, we estimated a separate rate for each year of the study, based on an approach that was previously used for mountain gorillas [7]. In each year, the estimated growth rate equaled: (N_births_ – N_deaths_) / Pop_avg_. Where N_births_ is the number of births during the year, N_deaths_ is the number of deaths, and Pop_avg_ is the average population size. To estimate the number of deaths, we assumed that each unexplained disappearance had a 50% probability of being caused by dispersal versus death. The population size was calculated as the average from the first day of each month. We ran a linear regression with one data point for each year of the study. The predictor variable was the year. The response variable was the estimated growth rate for the year. Each data point was weighted according to the population size, to avoid excessive influence from years with smaller sample sizes. Presumably the correlation would be weaker if we incorporated uncertainty about the fate of unexplained disappearances, rather than assuming a constant probability for all years (as in the growth rate calculation in the main text). The simplified analysis was already not significant, however, so a more complex approach does not seem necessary in this case. The average growth rate was also weighted according to the population size in each year.

As mentioned in the Discussion of the main text, the group density during this study was lower than the Virunga mountain gorillas. The group density for the Virungas was taken from Figure 3 of Caillaud (2020). The group density for Mbeli was based on a line-transect survey around the bai between May and July of 2006, which estimated a density of 0.87 weaned gorillas per km^2^ [20]. We calculated the group density during the survey as:

*StudyGroupDensity_2006_* = (*GorillaDensity_2006_*) x (*StudyGroups_2006_*) / (*StudyGorillas_2006_*)

= (0.87 gorillas/km^2^) x (16 groups) / (116 gorillas) = 0.12 groups/km^2^

Where *GorillaDensity_2006_* is the density of weaned gorillas as measured by the survey, *StudyGroups_2006_* is the number of groups in our study in June 2006, and *StudyGorillas_2006_* is the number of weaned gorillas in our study in June 2006. In other words, the group density equals the gorilla density, multiplied by the ratio of groups : gorillas.

We calculated the final (maximum) group density as:

*StudyGroupDensity_2020_* = *StudyGroupDensity_2006_* x (*StudyGroups_2020_*) / (*StudyGroups_2006_*)

= (0.12 groups_2006_/km^2^) x (27 groups_2020_) / (16 groups_2006_) = 0.20 groups_2020_/km^2^

Where *StudyGroups_2020_* is the number of groups in our study in May 2020, and the other terms are taken from the previous equation. In other words, the change in density from 2006 to 2020 is proportional to the corresponding change in the number of groups. The estimate of *StudyGroupDensity_2020_* does not specify an exclusive area for our study groups, which could be impossible because their collective home ranges have undoubtedly overlapped with some nonstudy groups. Nonetheless, the line-transect survey was centered around the bai, and it estimated a comparable number of weaned gorillas as our study, so its density estimate should be representative of our study.

***Methods for Section S4***

To compare the relative frequency of voluntary versus involuntary immigration among breeding groups, nonbreeding groups, and solitary males, we ran a GLMM with one data point for each female immigration from a study group. The response variable equaled “1” if the dispersal was voluntary, and “0” if it was not (disintegration). The response variable was a category variable to indicate whether the destination was a breeding group, nonbreeding group, or a solitary male. We included a random effect variable to control for multiple data points from the same females.

To compare the overall female immigration rate into breeding groups, nonbreeding groups, and solitary males, we ran a rate-based Chi-sq test [27]. We tallied the number of immigrations into each type of social unit, and the number of group-years that those units were observed. We calculated the expected number of immigrations into each type of social unit, based on the null hypothesis that the observed immigrations would be distributed in proportion to the number of years that each type of social unit was observed. We used a χ2 test to compare the expected versus actual number

of immigrations.

To estimate the age of groups that had already formed before they were observed, we defined the infants and juveniles in those groups as “probable offspring”, because they were unlikely to have immigrated. We found the earliest month in which a probable offspring was the oldest member of each group, except for the dominant male and adult females. In those group-months, we estimated the age of the group as 1.34 plus (1.13 times the age of the oldest probable offspring), where the parameters were taken from the analysis of groups whose formation was observed (Figure S-3b). We excluded groups in which a probable offspring was never the oldest member besides the dominant male and adult females.

Using only the groups whose formation was observed, we performed a linear regression to examine the correlation between the age of the group, versus the age of the oldest immature gorilla in the group. The regression used one data point for each month that the group was observed. We used those results to estimate the age of groups that had already formed when first observed. We incorporated those age estimates (along with the known ages of groups whose formation was observed) into survivorship calculations for the lifespan of groups. For each age “x”, “Nx” equaled the number of groups that were observed to reach the age, “dx” is the number of groups that ended at that age, and “cx” is the number of groups that were at the age when observations ended (censored data points). The probability that a group would “die” at age x was “qx”, which equaled dx / (Nx-(cx/2)). The probability that a group would survive at age x was Sx, which equalled 1-qx. The probably that a group would reach age x was lx, which equaled one for age zero, and lx-1 * Sx-1 for subsequent ages.

To compare the group size of breeding groups versus nonbreeding groups, we ran a GLMM with one data point for each month that each group was observed. The response variable was the size of the group on the first day of the month. The predictor variable indicated whether it was a breeding group or nonbreeding group. We included a control variable for temporal autocorrelation, and a random effect variable to control for multiple data points from the same groups.

To examine the overall variance in group size, we ran an ANOVA with one data point for each group on the first day of each year. The response variable was the average group size, and the predictor was a category variable for the group. The R2 from the ANOVA indicates the proportion of overall variance that occurred among groups, versus the variance within groups within each year.

To examine temporal variations in group size within groups, we ran another GLMM with one data point for each month that each group was observed. The response variable was the size of the group on the first day of the month. The predictor variable was the elapsed time since the group was formed. We included a control variable for temporal autocorrelation, and a random effect variable to control for multiple data points from the same groups. We ran a similar GLMM in which the predictor variable was the time until the group disintegrated. To examine temporal variations in the type of groups, we ran a similar pair of GLMM in which the predictor variable was the elapsed time since the group was formed, and the time until the group disintegrated. In both of those GLMM, the response variable equaled “1” when the group was a breeding group, and “0” when it was not.

***Methods for Section S5***

For each year from 1996 to 2015, we defined the “relative visitation frequency” as the cumulative number of times that a gorilla was observed at the bai, divided by the number of observation-hours. Visitation data was not available from 2016-2020. To estimate the rate of increase in the relative visitation frequency, we ran a linear regression with one data point for each year from 1996 to 2015. The response variable was the log transformation of the relative visitation frequency. The predictor variable was the year of the study. The slope of the line represents the average annual rate of increase. We ran one linear regression that focused on visited by identified gorillas in the demographic database, and a second regression that included visits by unidentified gorillas.

**Section S7. Tables for the Supporting Information**

Table S1.1 -- Life history parameters among female mountain gorillas at Karisoke and Bwindi, as well as female western gorillas at Mbeli. See Section S1 of the Supporting Information for citations of the source data for each parameter.

| parameter | Units | Karisoke | Bwindi | Mbeli |
| --- | --- | --- | --- | --- |
| immature mortality rate | deaths per immature-year | 0.087 | 0.093 | 0.119 |
| age of first reproduction | years | 8 | 8 | 10 |
| interbirth interval | months | 47.8 | 56.4 | 64.9 |
| adult mortality rate | deaths per adult-year | 0.03 | 0.032 | 0.036 |
| birth rate | births per adult female-year | 0.255 | 0.211 | 0.211 |
| %adults | % of all females | 61.9% | 65.7% | 62.8% |
| growth rate | Percent | 3.0% | 1.7% | 0.0% |

Table S2.1 -- Social units that entered the study via locational dispersal. The composition of those social units included previously unidentified infants (inf), juveniles (juv), subadults (sa), adult females (af), blackbacks (bb), young silverbacks (ysb), and adult silverbacks (sb). The “allnew” column shows the total for those previously unidentified gorillas. When they were identified, these immigrant groups also contained an additional 18 gorillas that had transferred from study groups, as indicated in the “transfers” column. In 5 of the 11 groups (shaded in grey), the only new gorilla was the silverback, so those cases could be considered locational dispersal of a solitary male who subsequently acquired a group in the area.

| Social unit | inf | juv | sa | af | bb | ysb | sb | allnew | transfers | total |
| --- | --- | --- | --- | --- | --- | --- | --- | --- | --- | --- |
| Bomba | 0 | 3 | 0 | 3 | 0 | 0 | 1 | 7 | 0 | 7 |
| Zulu | 1 | 0 | 0 | 5 | 0 | 0 | 1 | 7 | 0 | 7 |
| Grant | 1 | 0 | 0 | 2 | 0 | 0 | 1 | 4 | 0 | 4 |
| Atticus | 0 | 0 | 2 | 0 | 0 | 0 | 1 | 3 | 10 | 13 |
| BB | 0 | 0 | 1 | 0 | 1 | 0 | 0 | 2 | 0 | 2 |
| MM | 0 | 0 | 0 | 0 | 2 | 0 | 0 | 2 | 0 | 2 |
| James | 0 | 0 | 0 | 0 | 0 | 0 | 1 | 1 | 2 | 3 |
| Khan | 0 | 0 | 0 | 0 | 0 | 0 | 1 | 1 | 2 | 3 |
| Morpheus | 0 | 0 | 0 | 0 | 0 | 0 | 1 | 1 | 2 | 3 |
| Jacamo | 0 | 0 | 0 | 0 | 0 | 0 | 1 | 1 | 1 | 2 |
| Poko | 0 | 0 | 0 | 0 | 0 | 0 | 1 | 1 | 1 | 2 |
| Buchard | 0 | 0 | 0 | 0 | 0 | 1 | 0 | 1 | 0 | 1 |
| Conan | 0 | 0 | 0 | 0 | 0 | 0 | 1 | 1 | 0 | 1 |
| Geronimo | 0 | 0 | 0 | 0 | 0 | 0 | 1 | 1 | 0 | 1 |
| Kaspirov | 0 | 0 | 0 | 0 | 1 | 0 | 0 | 1 | 0 | 1 |
| Kyle | 0 | 0 | 0 | 0 | 0 | 1 | 0 | 1 | 0 | 1 |
| Lemmy | 0 | 0 | 0 | 0 | 0 | 0 | 1 | 1 | 0 | 1 |
| Munster | 0 | 0 | 0 | 0 | 0 | 1 | 0 | 1 | 0 | 1 |
| New Vidal | 0 | 0 | 0 | 0 | 0 | 1 | 0 | 1 | 0 | 1 |
| Orion | 0 | 0 | 0 | 0 | 0 | 1 | 0 | 1 | 0 | 1 |
| Sabinyo | 0 | 0 | 0 | 0 | 0 | 0 | 1 | 1 | 0 | 1 |
| Sulatalu | 0 | 0 | 0 | 0 | 0 | 0 | 1 | 1 | 0 | 1 |
| Ted | 0 | 0 | 0 | 0 | 0 | 0 | 1 | 1 | 0 | 1 |
| Tuba | 0 | 0 | 0 | 0 | 0 | 1 | 0 | 1 | 0 | 1 |
| Voldy | 0 | 0 | 0 | 0 | 0 | 1 | 0 | 1 | 0 | 1 |

Table S2.2 -- Influx, outflow, and internal transfers by subadult females and adult females. For each age class, the number of deaths was estimated as the number of female-years observed, multiplied by the mortality rate from the Leslie matrix calculations (deaths per female-year). The estimated outflow equals the reported number of unexplained disappearances, minus the estimated deaths. The total transfers from study groups equals the estimated outflow plus the number of observed transfers between study groups. The %outflow equals the estimated outflow, divided by the total transfers. The ratio of influx:outflow equals the observed influx, divided by the estimated outflow.

| parameter | subadults | adults |
| --- | --- | --- |
| mortality rate | 0.052 | 0.036 |
| female-years | 95.8 | 1338.0 |
| estimated deaths | 5 | 48 |
| disappearances | 8 | 73 |
| estimated outflow | 3 | 25 |
| internal transfers | 19 | 115 |
| total transfers | 22 | 140 |
| %outflow | 14% | 18% |
| Influx | 22 | 62 |
| influx:outflow | 7.4 | 2.5 |

Table S2.3 -- Composition of study groups when the dominant male died and the group disintegrated: infants (inf), juveniles (juv), subadults (sa), adult females (af), blackbacks (bb), young silverbacks (ysb), and adult silverbacks (sb). The juvenile and subadult categories are also combined for males (jsmale) and females (jsfemale), which exclude gorillas whose sex was unknown. Nonbreeding groups are shaded grey.

| Group | inf | juv | sa | af | bb | ysb | sb | total | jsmale | jsfemale |
| --- | --- | --- | --- | --- | --- | --- | --- | --- | --- | --- |
| Atticus | 0 | 2 | 2 | 2 | 7 | 3 | 1 | 17 | 2 | 1 |
| Emerson | 2 | 2 | 3 | 3 | 1 | 0 | 1 | 12 | 3 | 2 |
| Noodles | 2 | 0 | 4 | 2 | 1 | 2 | 1 | 12 | 3 | 1 |
| Clive | 2 | 3 | 0 | 4 | 0 | 0 | 1 | 10 | 2 | 1 |
| Duke | 2 | 3 | 1 | 3 | 0 | 0 | 1 | 10 | 1 | 1 |
| Bayleaf | 2 | 0 | 3 | 2 | 0 | 0 | 1 | 8 | 3 | 0 |
| Ob1 | 1 | 1 | 1 | 1 | 2 | 1 | 1 | 8 | 0 | 2 |
| Snowflake | 1 | 2 | 0 | 4 | 0 | 0 | 1 | 8 | 2 | 0 |
| FFF | 1 | 0 | 1 | 2 | 1 | 1 | 1 | 7 | 0 | 1 |
| Mosombo | 1 | 2 | 1 | 1 | 0 | 1 | 1 | 7 | 3 | 0 |
| Bones | 0 | 1 | 1 | 1 | 0 | 1 | 1 | 5 | 1 | 1 |
| Bear | 0 | 0 | 0 | 1 | 1 | 1 | 1 | 4 | 0 | 0 |
| Sangha | 0 | 1 | 0 | 1 | 0 | 0 | 1 | 3 | 1 | 0 |
| Sulatalu | 1 | 0 | 0 | 1 | 0 | 0 | 1 | 3 | 0 | 0 |
| TSB | 0 | 0 | 0 | 0 | 3 | 4 | 1 | 8 | 0 | 0 |
| George | 0 | 1 | 1 | 0 | 0 | 1 | 1 | 4 | 1 | 1 |
| James | 0 | 0 | 0 | 0 | 0 | 1 | 1 | 2 | 0 | 0 |
| Travis | 0 | 0 | 0 | 0 | 0 | 1 | 1 | 2 | 0 | 0 |
| total | 15 | 18 | 18 | 28 | 16 | 17 | 18 | 130 | 22 | 11 |

Table S2.4 -- Fates of gorillas in groups when the dominant male died: infants (inf), juveniles (juv), subadults (sa), adult females (af), blackbacks (bb), young silverbacks (ysb), and adult silverbacks (sb). The juvenile and subadult categories are also combined for males (jsmale) and females (jsfemale), which exclude gorillas whose sex was unknown.

| fate | inf | juv | sa | af | bb | ysb | sb | total | jsmale | jsfemale |
| --- | --- | --- | --- | --- | --- | --- | --- | --- | --- | --- |
| join breeding group | 1 | 4 | 3 | 9 | 0 | 0 | 0 | 17 | 5 | 2 |
| join nonbreeding group | 5 | 5 | 7 | 7 | 5 | 7 | 0 | 36 | 9 | 3 |
| join solitary male | 0 | 2 | 4 | 3 | 7 | 2 | 0 | 18 | 3 | 2 |
| become solitary male | 0 | 0 | 0 | 0 | 3 | 6 | 0 | 9 | 0 | 0 |
| temporary disappearance | 1 | 0 | 3 | 2 | 1 | 0 | 0 | 7 | 2 | 1 |
| permanent disappearance | 8 | 7 | 1 | 7 | 0 | 2 | 18 | 43 | 3 | 3 |
| total | 15 | 18 | 18 | 28 | 16 | 17 | 18 | 130 | 22 | 11 |

Table S4.1 -- Summary of the study groups. How the groups started and ended, unless they were present at the beginning or end of the study. Percentage of time that they were breeding groups. The age of the group when first observed, along with the number of group-years, gorilla-years, and adult female-years observed. The number of births, immigrations, and disappearances (which could be deaths or emigration). Percentage of infants that survived to reach age four. The average, minimum and maximum number of gorillas in the group.

|  | How | How | breeding | first | group | gorilla | AF |  | infant |  | death & | group size | | |
| --- | --- | --- | --- | --- | --- | --- | --- | --- | --- | --- | --- | --- | --- | --- |
| group | Started | ended | group% | age | years | years | years | births | surv% | influx | outflow | avg | min | max |
| Atticus | Appear | disintegrate | 96% |  | 2.2 | 34.6 | 4.1 | 0 | 0% | 8 | 21 | 16.0 | 11 | 18 |
| Baco | Fission | present | 19% |  | 3.1 | 16.0 | 0.6 | 0 | 0% | 0 | 1 | 5.2 | 5 | 6 |
| Bayleaf | Formed | disintegrate | 100% | 0.0 | 12.0 | 100.8 | 47.2 | 7 | 57% | 6 | 16 | 8.4 | 2 | 13 |
| BB | Appear | disintegrate | 0% |  | 1.9 | 3.8 | 0.0 | 0 | 0% | 0 | 1 | 2.0 | 2 | 2 |
| Bear | Formed | disintegrate | 8% | 0.0 | 4.3 | 18.8 | 0.3 | 0 | 0% | 1 | 6 | 4.3 | 4 | 5 |
| Bomba | appear | present | 19% | 8.1 | 13.3 | 60.8 | 6.2 | 1 | 0% | 0 | 4 | 4.6 | 4 | 7 |
| Bones | present | disintegrate | 100% |  | 3.2 | 15.8 | 3.2 | 0 | 0% | 0 | 5 | 5.0 | 4 | 5 |
| Boris | formed | attrition | 100% | 0.1 | 2.3 | 6.3 | 2.3 | 1 | 0% | 0 | 3 | 2.7 | 2 | 3 |
| Buster | formed | attrition | 100% | 0.0 | 3.1 | 6.2 | 3.1 | 0 | 0% | 0 | 2 | 2.0 | 2 | 2 |
| Clive | present | disintegrate | 100% | 2.5 | 5.6 | 42.5 | 20.3 | 3 | 67% | 3 | 14 | 7.6 | 5 | 10 |
| Conan | appear | present | 100% | 0.6 | 19.1 | 200.7 | 75.4 | 14 | 64% | 6 | 13 | 10.5 | 2 | 13 |
| Coriander | formed | present | 100% | 0.1 | 13.5 | 161.7 | 77.6 | 17 | 58% | 12 | 14 | 12.0 | 2 | 17 |
| Courtney | formed | fission | 0% | 0.0 | 2.1 | 21.3 | 0.0 | 0 | 0% | 5 | 14 | 10.2 | 8 | 13 |
| Darzee | formed | present | 100% | 0.1 | 4.8 | 31.8 | 18.8 | 3 | 100% | 4 | 1 | 6.7 | 2 | 8 |
| Dill | formed | present | 100% | 0.1 | 6.7 | 37.3 | 24.5 | 1 | 100% | 3 | 0 | 5.6 | 2 | 6 |
| Duke | present | disintegrate | 100% |  | 4.1 | 39.7 | 12.3 | 2 | 0% | 0 | 10 | 9.7 | 7 | 11 |
| Dwayne | formed | present | 72% | 0.1 | 24.0 | 240.1 | 83.6 | 16 | 75% | 13 | 28 | 10.0 | 2 | 16 |
| Emerson | present | disintegrate | 93% | 5.9 | 7.1 | 86.7 | 33.0 | 4 | 50% | 0 | 16 | 12.2 | 3 | 14 |
| FFF | present | disintegrate | 100% |  | 1.6 | 7.3 | 2.1 | 0 | 0% | 0 | 7 | 4.6 | 3 | 7 |
| Frank | formed | attrition | 100% | 0.1 | 1.4 | 5.3 | 2.6 | 1 | 0% | 1 | 4 | 3.7 | 3 | 5 |
| George | formed | disintegrate | 99% | 0.0 | 18.8 | 156.1 | 58.7 | 12 | 58% | 14 | 28 | 8.3 | 2 | 14 |
| Grant | appear | attrition | 100% | 4.7 | 6.3 | 22.1 | 8.8 | 1 | 0% | 0 | 4 | 3.5 | 2 | 4 |
| Gretsky | formed | present | 100% | 0.1 | 16.4 | 214.3 | 99.7 | 23 | 58% | 13 | 29 | 13.1 | 3 | 18 |
| Iook | formed | present | 0% | 0.1 | 0.8 | 1.7 | 0.0 | 0 | 0% | 0 | 0 | 2.0 | 2 | 2 |
| Isaak | formed | present | 100% | 0.0 | 12.3 | 58.6 | 27.7 | 2 | 100% | 6 | 5 | 4.8 | 2 | 7 |
| Jacamo | appear | present | 100% | 0.7 | 8.8 | 130.7 | 67.3 | 18 | 50% | 14 | 13 | 14.9 | 2 | 21 |

Table S4.1 continued.

|  | how | how | breeding | first | group | gorilla | AF |  | infant |  | death & | group size | | |
| --- | --- | --- | --- | --- | --- | --- | --- | --- | --- | --- | --- | --- | --- | --- |
| group | started | ended | group% | age | years | years | years | births | surv% | influx | outflow | avg | min | max |
| James | appear | disintegrate | 41% |  | 2.3 | 12.1 | 0.9 | 0 | 0% | 8 | 10 | 5.4 | 2 | 11 |
| Khan | appear | present | 82% | 0.0 | 23.9 | 243.8 | 92.2 | 16 | 56% | 22 | 34 | 10.2 | 3 | 18 |
| Kotick | formed | attrition | 100% | 0.0 | 1.9 | 8.1 | 3.8 | 0 | 0% | 5 | 6 | 4.2 | 2 | 6 |
| Lords | fission | present | 0% |  | 3.1 | 26.8 | 0.0 | 0 | 0% | 0 | 6 | 8.7 | 6 | 12 |
| Louis | formed | present | 0% | 0.1 | 0.9 | 1.8 | 0.0 | 0 | 0% | 0 | 0 | 2.0 | 2 | 2 |
| Lyle | formed | present | 100% | 0.1 | 4.7 | 20.9 | 8.3 | 2 | 0% | 5 | 0 | 4.5 | 2 | 9 |
| Morpheus | appear | present | 57% | 0.7 | 16.6 | 135.5 | 37.3 | 8 | 75% | 12 | 15 | 8.2 | 3 | 14 |
| Mosombo | present | disintegrate | 37% | 1.3 | 6.5 | 22.5 | 2.4 | 0 | 0% | 8 | 10 | 3.5 | 2 | 7 |
| Mowgli | formed | present | 100% | 0.0 | 0.4 | 1.9 | 1.1 | 0 | 0% | 2 | 0 | 4.6 | 3 | 5 |
| Munster | formed | present | 100% | 0.0 | 9.4 | 57.8 | 25.1 | 4 | 100% | 1 | 1 | 6.1 | 3 | 7 |
| New Vidal | formed | attrition | 100% | 0.0 | 12.1 | 44.6 | 23.1 | 3 | 50% | 5 | 7 | 3.7 | 2 | 6 |
| Noodles | present | disintegrate | 79% |  | 8.4 | 79.0 | 10.5 | 1 | 100% | 13 | 22 | 9.4 | 6 | 12 |
| Ob1 | present | disintegrate | 100% | 9.6 | 11.8 | 119.8 | 30.9 | 7 | 14% | 6 | 24 | 10.2 | 6 | 12 |
| Orion | formed | present | 97% | 0.0 | 2.7 | 11.3 | 7.0 | 3 | 0% | 3 | 0 | 4.3 | 2 | 8 |
| Poko | appear | present | 100% | 0.4 | 6.8 | 55.2 | 29.3 | 5 | 100% | 9 | 5 | 8.1 | 2 | 11 |
| Saha | formed | present | 100% | 0.1 | 7.0 | 50.2 | 21.5 | 8 | 50% | 6 | 12 | 7.2 | 2 | 10 |
| Sangha | present | disintegrate | 100% | 2.6 | 22.5 | 133.0 | 48.3 | 8 | 38% | 0 | 14 | 5.9 | 2 | 9 |
| Sassoon | present | attrition | 100% | 1.4 | 9.3 | 74.5 | 29.8 | 6 | 33% | 6 | 16 | 8.0 | 2 | 12 |
| Scout | formed | present | 100% | 0.0 | 7.3 | 50.4 | 26.9 | 2 | 100% | 5 | 1 | 7.0 | 2 | 9 |
| Snowflake | present | disintegrate | 100% | 3.3 | 4.3 | 31.6 | 15.9 | 4 | 0% | 1 | 13 | 7.4 | 2 | 10 |
| Stockwell | formed | disintegrate | 74% | 0.1 | 12.0 | 54.3 | 20.7 | 3 | 100% | 3 | 7 | 4.5 | 2 | 7 |
| Sulatalu | formed | disintegrate | 100% | 0.0 | 6.5 | 17.8 | 7.3 | 3 | 0% | 2 | 6 | 2.7 | 2 | 4 |
| Tarragon | formed | attrition | 7% | 0.0 | 12.8 | 73.3 | 0.9 | 0 | 0% | 5 | 17 | 5.7 | 2 | 12 |
| Tikki | formed | present | 100% | 0.0 | 2.1 | 4.3 | 2.3 | 0 | 0% | 1 | 1 | 2.1 | 2 | 3 |
| Travis | present | disintegrate | 30% |  | 5.3 | 16.0 | 1.6 | 0 | 0% | 0 | 5 | 3.0 | 2 | 5 |
| TSB | present | disintegrate | 24% |  | 13.3 | 77.5 | 3.7 | 2 | 0% | 8 | 17 | 5.8 | 2 | 10 |
| Tuba | formed | present | 95% | 0.0 | 10.3 | 95.9 | 51.8 | 14 | 0% | 8 | 14 | 9.4 | 3 | 13 |
| Vidal | formed | present | 100% | 0.1 | 11.7 | 114.7 | 46.2 | 11 | 86% | 7 | 6 | 9.8 | 3 | 15 |
| Zulu | appear | present | 100% | 1.4 | 19.8 | 209.3 | 75.3 | 17 | 56% | 5 | 23 | 10.6 | 7 | 15 |

Table S4.2 -- Number of transitions between the types of social units, such as changes from solitary males to breeding groups (sm>bg) or nonbreeding groups (sm>nbg). Transitions occurred through voluntary dispersal or involuntary dispersal (group disintegrations), which cannot always be distinguished if the immigrants come from a non-study group. Nonbreeding groups can also become breeding groups when a female reaches adulthood. The total number of transitions may not equal the sum of those mechanisms, because multiple mechanisms sometimes occurred during the same transition. For example, a solitary male could have acquired gorillas via both voluntary and involuntary dispersal during an interval when he was not observed.

| mechanisms | change in type of social unit | | |
| --- | --- | --- | --- |
| to gain gorillas | sm>bg | sm>nbg | nbg>bg |
| voluntary dispersal | 18 | 3 | 3 |
| involuntary dispersal | 2 | 3 | 1 |
| from non-study group | 8 | 4 | 3 |
| female reaches age 10 | 0 | 0 | 4 |
| total | 22 | 7 | 11 |
|  |  |  |  |
|  |  |  |  |
| mechanisms | change in type of social unit | | |
| to lose gorillas | nbg>sm | bg>nbg | bg>sm |
| voluntary dispersal | 1 | 7 | 9 |
| disappearance | 0 | 8 | 1 |
| total | 1 | 15 | 10 |

Table S4.3 -- Number of immigrations by adult and subadult females to join breeding groups, nonbreeding groups, and solitary males. Immigration can be voluntary (vol) or involuntary (invol), which cannot be distinguished if the immigrants come from an unknown group (unk). The immigration rate equals the total number of immigrations (tot), divided by the number of years that the social units were observed (obs-yrs).

| social unit | obs-yrs | vol | invol | unk | tot | rate |
| --- | --- | --- | --- | --- | --- | --- |
| breeding group | 365.1 | 76 | 9 | 85 | 170 | 0.466 |
| nonbreeding group | 73.9 | 6 | 4 | 4 | 14 | 0.189 |
| solitary male | 278.3 | 23 | 4 | 13 | 40 | 0.144 |
| Total | 717.3 | 105 | 17 | 102 | 224 | 0.312 |

Table S4.4 – Summary of each solitary phase by each male. Of the 85 solitary phases, five were present when observation started (6%), five began through involuntary dispersal during a group disintegration (6%), 52 began by voluntary emigration (61%), eleven began through attrition when a dominant male lost his other group members (13%), and twelve solitary males appeared during the study (14%). Six of the solitary phases ended when the male joined another social unit (7%), 33 ended when the male formed a group (39%), 25 of the solitary males disappeared (29%), and 21 were still present when the study ended (25%). This tabulation includes only solitary phases that were observed on the first day of at least one month.

| solitary | social | solitary | how | how | start | Stop | duration |
| --- | --- | --- | --- | --- | --- | --- | --- |
| male | unit | phase | started | ended | age | age | months |
| Aragon | Aragon | Aragon-1 | emigration | present | 22.2 | 22.4 | 4 |
| Bayleaf | Bayleaf | Bayleaf-1 | emigration | form group | 16.1 | 18.9 | 35 |
| Bear | Bear | Bear-1 | present | form group | 20.8 | 27.8 | 85 |
| Bggins | Bggins | Bggins-1 | emigration | present | 17.2 | 18.4 | 16 |
| Bird | Bird | Bird-1 | emigration | disappear | 14.8 | 14.9 | 3 |
| Bogplant | Bogplant | Bogplant-1 | emigration | present | 16.3 | 29.3 | 157 |
| Bond | Bond | Bond-1 | emigration | disappear | 16.8 | 17.6 | 10 |
| Boris | BB | Boris-1 | disintegration | immigration | 14.5 | 14.5 | 1 |
| Boris | Boris | Boris-2 | emigration | form group | 15.2 | 23.8 | 105 |
| Boris | Boris | Boris-3 | attrition | form group | 24.0 | 24.2 | 3 |
| Boris | Boris | Boris-4 | attrition | present | 26.5 | 29.3 | 35 |
| Buchard | Buchard | Buchard-1 | appear | disappear | 17.0 | 21.3 | 53 |
| Buster | Buster | Buster-1 | emigration | form group | 15.9 | 20.4 | 55 |
| Buster | Buster | Buster-2 | attrition | form group | 20.6 | 20.7 | 2 |
| Buster | Buster | Buster-3 | attrition | present | 23.7 | 25.3 | 20 |
| Coriander | Coriander | Coriander-1 | emigration | form group | 15.0 | 17.4 | 29 |
| Costa | Costa | Costa-1 | emigration | disappear | 17.4 | 19.1 | 21 |
| Courtney | Duke | Courtney-1 | disintegration | immigration | 9.4 | 9.8 | 6 |
| Courtney | Courtney | Courtney-2 | emigration | form group | 16.4 | 24.4 | 97 |
| Custer | Custer | Custer-1 | emigration | present | 17.0 | 18.4 | 17 |
| Darzee | Darzee | Darzee-1 | emigration | form group | 14.1 | 17.8 | 45 |
| Dill | Dill | Dill-1 | emigration | form group | 16.4 | 21.6 | 63 |
| Djino | Djino | Djino-1 | emigration | disappear | 15.8 | 16.8 | 12 |
| Dwayne | Dwayne | Dwayne-1 | present | form group | 18.0 | 18.3 | 5 |
| Dylan | Dylan | Dylan-1 | emigration | disappear | 15.1 | 19.0 | 47 |
| Elasser | Elasser | Elasser-1 | emigration | present | 19.3 | 19.5 | 4 |
| Frank | Frank | Frank-1 | emigration | form group | 15.2 | 18.2 | 37 |
| Frank | Frank | Frank-2 | attrition | disappear | 19.7 | 20.0 | 5 |
| Gandalf | Gandalf | Gandalf-1 | emigration | present | 16.8 | 17.5 | 10 |
| George | George | George-1 | present | form group | 20.8 | 22.3 | 19 |
| Geronimo | Geronimo | Geronimo-1 | appear | disappear | 20.1 | 21.2 | 15 |
| Gimle | Gimle | Gimle-1 | emigration | present | 16.2 | 18.4 | 27 |
| Grant | Grant | Grant-1 | attrition | disappear | 26.4 | 26.6 | 3 |
| Gretsky | Gretsky | Gretsky-1 | emigration | form group | 16.1 | 19.4 | 41 |

Table S4.3 – continued

| solitary | social | solitary | how | how | start | Stop | duration |
| --- | --- | --- | --- | --- | --- | --- | --- |
| male | unit | phase | started | ended | age | age | months |
| Homer | Homer | Homer-1 | emigration | disappear | 15.4 | 15.4 | 1 |
| Iook | Iook | Iook-1 | emigration | form group | 18.0 | 25.4 | 90 |
| Isaak | Isaak | Isaak-1 | emigration | form group | 16.7 | 21.9 | 64 |
| Jaica | Jaica | Jaica-1 | emigration | disappear | 17.5 | 17.9 | 6 |
| Kaspirov | Kaspirov | Kaspirov-1 | appear | present | 12.1 | 24.7 | 153 |
| Kingsley | James | Kingsley-1 | disintegration | immigration | 15.7 | 16.2 | 8 |
| Kingsley | Kingsley | Kingsley-2 | emigration | present | 18.5 | 26.3 | 95 |
| Kotick | Kotick | Kotick-1 | emigration | form group | 18.0 | 20.0 | 25 |
| Kotick | Kotick | Kotick-2 | attrition | present | 22.0 | 22.2 | 4 |
| Kurtz | Kurtz | Kurtz-1 | emigration | disappear | 16.2 | 21.3 | 62 |
| Lemmy | Lemmy | Lemmy-1 | appear | disappear | 30.0 | 36.9 | 84 |
| Louis | Louis | Louis-1 | emigration | form group | 15.1 | 21.5 | 78 |
| Lyle | Lyle | Lyle-1 | emigration | form group | 15.2 | 18.9 | 45 |
| Mahale | Mahale | Mahale-1 | emigration | present | 19.7 | 25.9 | 75 |
| Mondele | Mondele | Mondele-1 | emigration | disappear | 12.7 | 16.4 | 46 |
| Monty | Monty | Monty-1 | emigration | present | 15.4 | 24.9 | 115 |
| Mosidole | Mosidole | Mosidole-1 | present | disappear | 20.8 | 21.3 | 7 |
| Mowgli | Mowgli | Mowgli-1 | emigration | form group | 16.1 | 19.7 | 44 |
| Munster | Munster | Munster-1 | appear | form group | 16.8 | 21.5 | 57 |
| Ndjeke | Ndjeke | Ndjeke-1 | emigration | present | 17.3 | 22.1 | 59 |
| Ndoki | Ndoki | Ndoki-1 | emigration | present | 19.9 | 25.2 | 65 |
| New Vidal | New Vidal | New Vidal-1 | appear | form group | 15.8 | 19.3 | 43 |
| New Vidal | New Vidal | New Vidal-2 | attrition | form group | 19.9 | 21.7 | 23 |
| Orion | Orion | Orion-1 | appear | form group | 15.7 | 19.7 | 49 |
| Peapod | Peapod | Peapod-1 | emigration | present | 16.4 | 20.7 | 53 |
| Pepper | Pepper | Pepper-1 | emigration | disappear | 16.3 | 17.6 | 16 |
| Rudyard | Rudyard | Rudyard-1 | emigration | disappear | 15.9 | 18.1 | 27 |
| Sabinyo | Sabinyo | Sabinyo-1 | appear | disappear | 20.1 | 20.6 | 8 |
| Saha | Saha | Saha-1 | emigration | form group | 18.3 | 24.1 | 71 |
| Sassoon | Sassoon | Sassoon-1 | attrition | disappear | 40.0 | 47.7 | 93 |
| Scout | Scout | Scout-1 | emigration | form group | 16.5 | 18.7 | 27 |

Table S4.3 – continued again

| solitary | social | solitary | how | how | start | stop | duration |
| --- | --- | --- | --- | --- | --- | --- | --- |
| male | unit | phase | started | ended | age | age | months |
| Sembe | Sangha | Sembe-1 | disintegration | disappear | 5.8 | 6.1 | 4 |
| Smeagol | Smeagol | Smeagol-1 | emigration | present | 18.8 | 19.5 | 9 |
| Stockwell | Stockwell | Stockwell-1 | emigration | form group | 17.5 | 22.8 | 65 |
| Sulatalu | Sulatalu | Sulatalu-1 | appear | form group | 20.0 | 21.2 | 15 |
| Tarragon | Tarragon | Tarragon-1 | emigration | form group | 16.8 | 22.6 | 70 |
| Tarragon | Tarragon | Tarragon-2 | attrition | form group | 23.6 | 24.2 | 8 |
| Tarragon | Tarragon | Tarragon-3 | attrition | present | 36.2 | 38.0 | 23 |
| Tavi | Tavi | Tavi-1 | emigration | disappear | 17.0 | 18.0 | 13 |
| Ted | Ted | Ted-1 | appear | disappear | 20.0 | 20.1 | 2 |
| Tikki | Tikki | Tikki-1 | emigration | form group | 15.4 | 18.8 | 42 |
| Tuba | Tuba | Tuba-1 | appear | form group | 17.6 | 20.1 | 31 |
| Uhura | Uhura | Uhura-1 | emigration | disappear | 15.1 | 16.5 | 18 |
| Ulysses | Ulysses | Ulysses-1 | emigration | immigration | 12.8 | 17.0 | 51 |
| Vianey | Bayleaf | Vianey-1 | disintegration | immigration | 8.8 | 8.8 | 1 |
| Vidal | Vidal | Vidal-1 | emigration | form group | 15.0 | 21.7 | 81 |
| Vince | Vince | Vince-1 | present | disappear | 20.7 | 34.3 | 165 |
| Voldy | Voldy | Voldy-1 | appear | immigration | 16.4 | 16.4 | 1 |
| Waldorf | Waldorf | Waldorf-1 | emigration | disappear | 13.1 | 14.0 | 12 |
| Whisky | Whisky | Whisky-1 | emigration | present | 19.2 | 19.5 | 4 |

**Section S8. Graphs for the Supporting Information**

Figure S1.1 -- Survivorship curves for female mountain gorillas at Bwindi and Karisoke, as well as female western gorillas at Mbeli. Data are taken from [28].


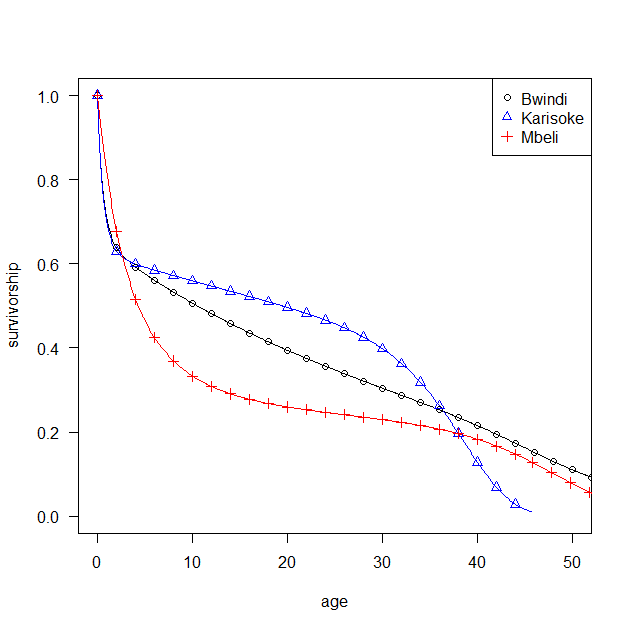


Figure S1.2 -- Differences in life history parameters among female mountain gorillas at Karisoke and Bwindi, as well as western gorillas at Mbeli. To provide a consistent basis for comparison, each parameter has been normalized by dividing by the value for Karisoke. Table S1 shows the data without normalization. In the legend, the parameters are listed in the predicted order of changes when a population density increases towards its carrying capacity: higher mortality rates for immatures, followed by a later age of first reproduction, then lower reproductive rates (e.g., longer interbirth intervals), and finally higher mortality rates for adults [4]. Only two data points for Mbeli (and none for Bwindi) are in the same order as those predictions.


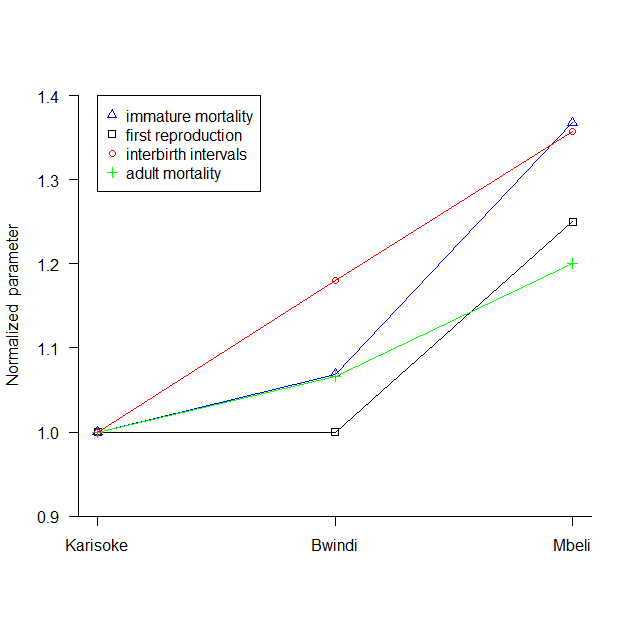


Figure S1.3 -- Fast-slow continuum for life history parameters among female mountain gorillas at Bwindi and Karisoke, as well as western gorillas at Mbeli. Across all ages, the mortality rate equals the number of female deaths, divided by the number of female-years. The birth rate equals the number of females born, divided by the number of all female-years (not just adult females). The diagonal life represents a growth rate of zero, because the rates of deaths and births are equal. Positive growth rates are above the diagonal, and negative rates are below. The populations at Karisoke, Bwindi, and Mbeli are indicated by the points labeled “K’, “B”, and “M”, respectively. In Figure S3a, the blue dashed lines illustrate how the Karisoke growth rate could reach zero exclusively through increased mortality rates (horizontal line to Km), or exclusively through reduced birth rates (vertical line to Kb). The red dashed lines show the corresponding scenarios for Bwindi. In Figure S3b, the dotted lines show equal changes in the rates of deaths and births. The solid blue line illustrates increased mortality and births, as might be expected with greater infanticide.


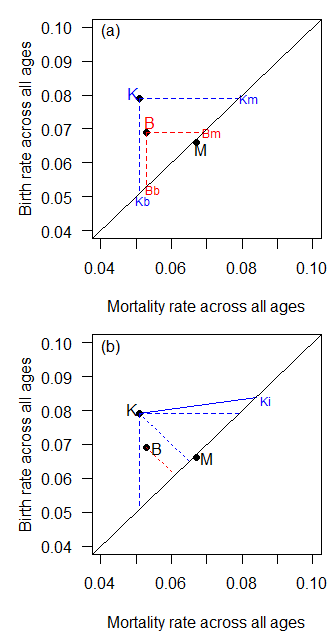


Figure S2.1 -- Cumulative age distribution of subordinate males who disappeared (x-marks), emigrated (triangles), or were still subordinate in a group when last observed (squares). Each data point represents one gorilla who was observed since becoming a blackback (at age 11). For example, approximately 67% of the unexplained disappearances by subordinate males had occurred by age 15.3 (dashed lines).


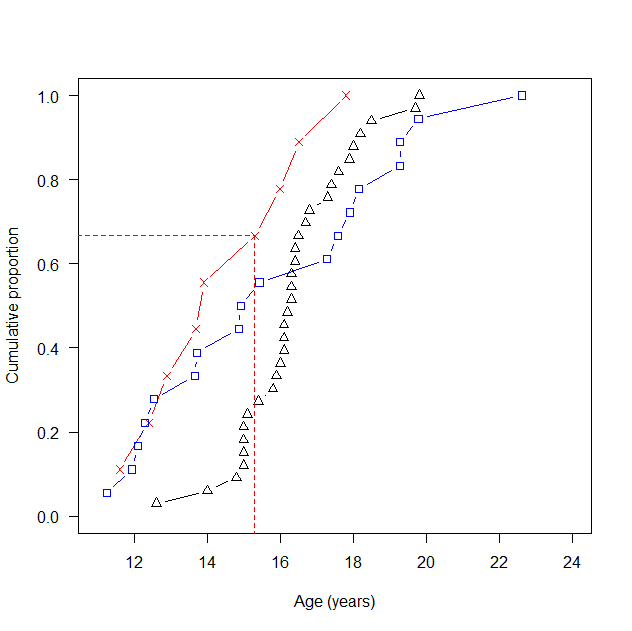


Figure S2.2 -- Cumulative distribution of male emigrants who disappeared (x-marks), acquired a group (circles), or were still solitary when last observed (inverted triangles). Each data point represents one gorilla who emigrated from a study group. For example, approximately 44% of the unexplained disappearances by subordinate males had occurred within one year after their emigration (dashed lines).


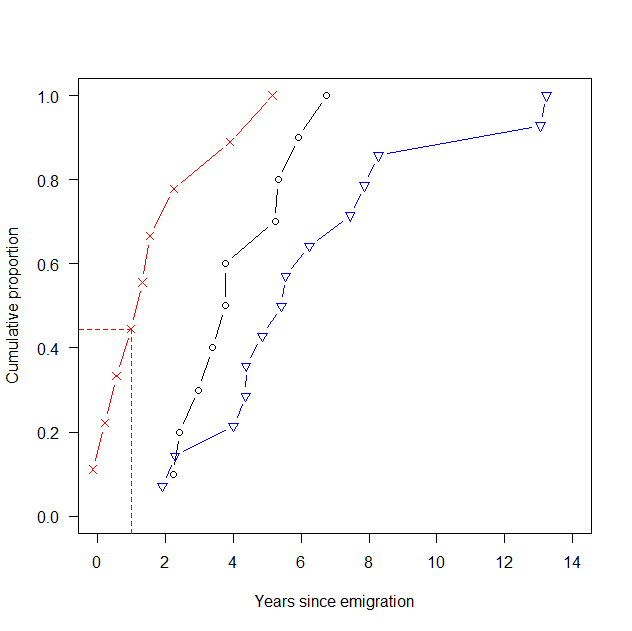


Figure S3.1 – Growth rate during each year of the study, based on an approach that was previously used for mountain gorillas (Caillaud et al, 2020). The estimated growth rates are not significantly correlated with the year (R^2^ = 0.048, F_23,1_ = 1.16, p = 0.29). The dashed line represents a stable population with a growth rate equal to zero.


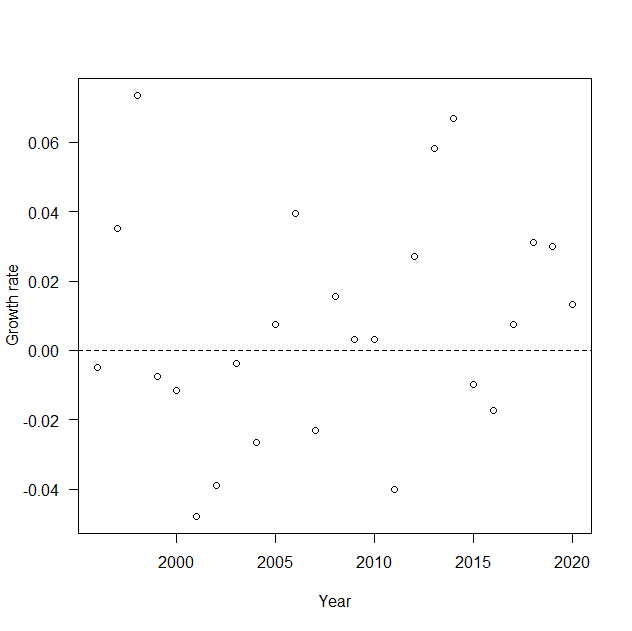


Figure S4.1 – Cumulative number of different mates for each adult female, versus her cumulative number of births. Each data point represents one combination of those two variables. The size of the data point reflects the number of females who have been observed with that combination (e.g., the largest circle represents females who have been observed for only one birth, and therefore have only one mate). The mate is assumed to be the silverback in the group where the female gave birth (Breuer 2009). The solid line is from a linear regression with one data point for each female, and the dashed lines are 95% prediction intervals.


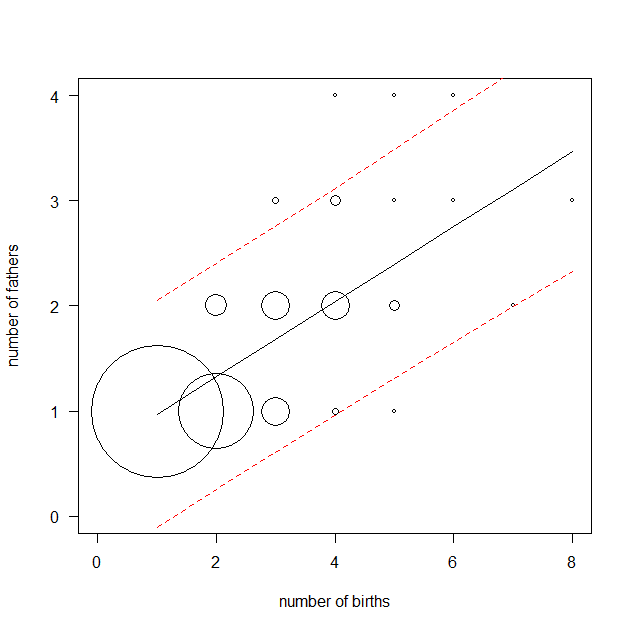


Figure S4.2 -- Age of the study groups versus the age of the oldest gorilla that was born in the group (known offspring). Each data point represents the conditions at the beginning of a month for a group that formed during the study. The correlation was used to estimate the age of groups that did not form during the study, based on the age of their oldest “probable offspring”. We defined probable offspring as the infants and juveniles in those groups, because they were unlikely to have immigrated.


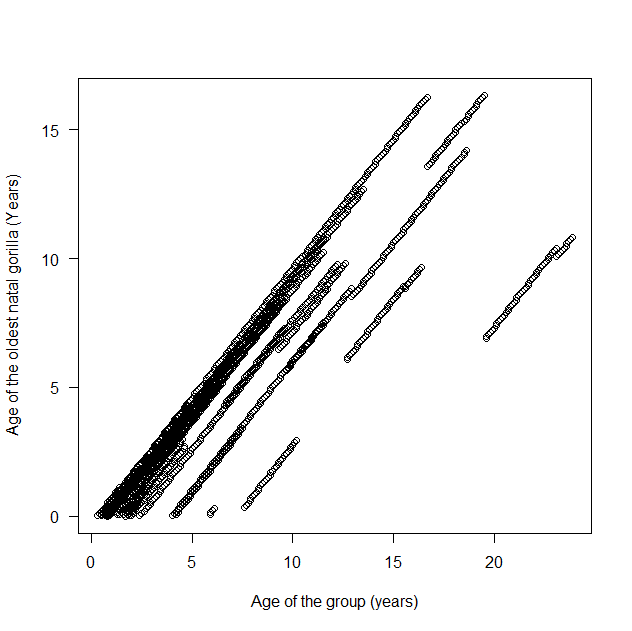


Figure S4.3 -- Survivorship curves for the estimated lifespan of groups. The black dots are based on groups that were observed since they were formed. The red line includes estimated ages for groups that formed outside the study. Both estimates include censorship of groups that were still present when observations ended.


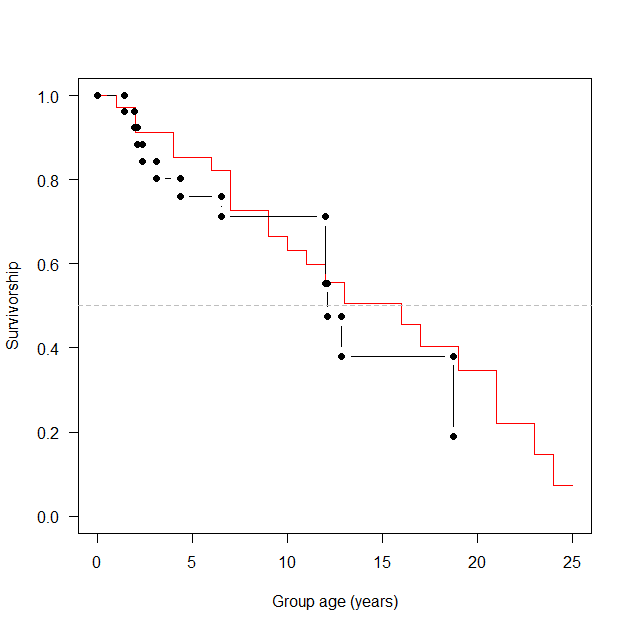


Figure S4.4 – Group size distribution for breeding groups (blue triangles), nonbreeding groups (red x-marks), and all groups combined (black circles). Breeding groups are significantly larger than nonbreeding groups (N = 5449 data points in 55 groups, Chi-sq = 9312, p < 0.001).


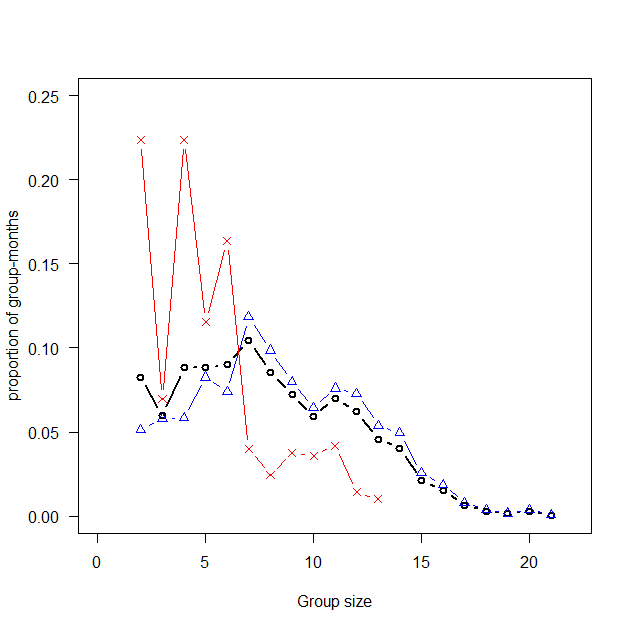


Figure S4.5 – Group size versus the time since the group began (a), and versus the time until the group disintegrated (b). The correlation is significantly positive for the time since the group began (N = 2458 data points in 29 groups, Chi-sq = 1132, p < 0.001), and for the time until the group disintegrated (N = 1627 data points in 19 groups, Chi-sq = 1043, p < 0.001). Dashed lines show 95% prediction intervals.


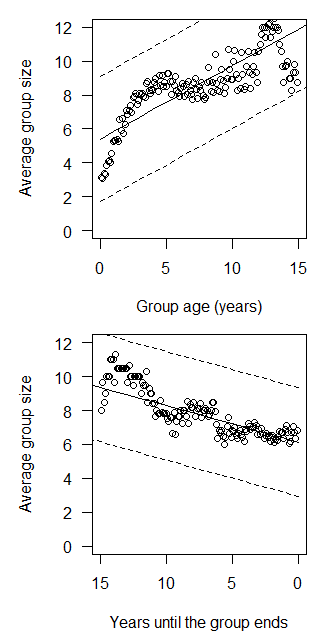


Figure S5.1 -- Temporal variations in the relative visitation frequency during observations at the bai. Solid lines are the predictions from a linear regression, and dashed lines are the 95% prediction intervals. The y-axes are on a log‑scale, so the slope of the regression lines indicate that the relative visitation frequency increased by 2.57% per year for identified gorillas in the study population (a), versus 2.52% for all gorillas including those that were not identified during a visit (b).


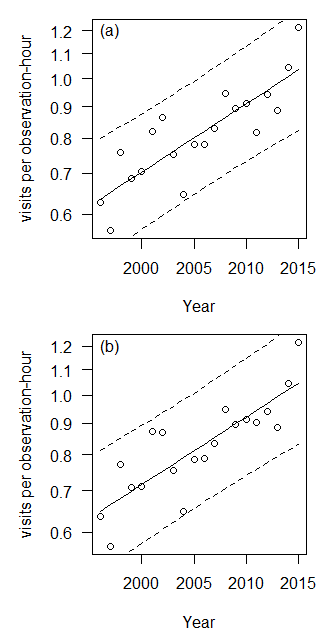


**Section S9. References for the Supporting Information**

1. Robbins MM, Gray M, Fawcett KA, Nutter FB, Uwingeli P, Mburanumwe I, et al. Extreme conservation leads to recovery of the Virunga mountain gorillas. Plos One. 2011;6(6):e19788. PubMed PMID: WOS:000291611500004.

2. Robbins MM, Gray M, Kagoda E, Robbins AM. Population dynamics of the Bwindi mountain gorillas. Biological Conservation. 2009;142(12):2886-95. PubMed PMID: BIOSIS:PREV201000039506.

3. Caswell H. Matrix population models. Sunderland, MA: Sinauer Associates; 2001. 713 p.

4. Eberhardt LL. A paradigm for population analysis of long-lived vertebrates. Ecology. 2002;83(10):2841-54. PubMed PMID: ISI:000179348800017.

5. Charnov EL. Life history invariants. Oxford: Oxford University Press; 1993. 168 p.

6. Robbins MM, Akantorana M, Arinaitwe J, Richardson J, Breuer T, Manguette M, et al. Comparative Life History Patterns of Female Gorilla. in review.

7. Caillaud D, Eckardt W, Vecellio V, Ndagijimana F, Mucyo JP, Hirwa JP, et al. Violent encounters between social units hinder the growth of a high-density mountain gorilla population. Science Advances. 2020;6(45). doi: 10.1126/sciadv.aba0724. PubMed PMID: WOS:000587544300006.

8. Stokes EJ, Parnell RJ, Olejniczak C. Female dispersal and reproductive success in wild western lowland gorillas (*Gorilla gorilla gorilla*). Behavioral Ecology and Sociobiology. 2003;54(4):329-39. PubMed PMID: ISI:000185091700002.

9. Kappeler PM, Pereira ME, van Schaik C. Primate life histories and socioecology. In: Kappeler PM, editor. Primate Life Histories and Socioecology. Cambridge: Cambridge University Press; 2003. p. 1-24.

10. Watts DP. The influence of male mating tactics on habitat use in mountain gorillas (*Gorilla gorilla beringei*). Primates. 1994;35(1):35-47. PubMed PMID: ISI:A1994MU06800004.

11. Caillaud D, Ndagijimana F, Giarrusso AJ, Vecellio V, Stoinski TS. Mountain gorilla ranging patterns: Influence of group size and group dynamics. American Journal of Primatology. 2014;76(8):730-46. PubMed PMID: WOS:000339478700003.

12. Grueter CC, Robbins AM, Abavandimwe D, Vecellio V, Ndagijimana F, Stoinski TS, et al. Quadratic relationships between group size and foraging efficiency in a herbivorous primate. Scientific Reports [Internet]. 2018 Nov; 8:[16718 p.]. Available from: <Go to ISI>://WOS:000449944500006.

13. Hagemann L, Arandjelovic M, Robbins MM, Deschner T, Lewis M, Froese G, et al. Long-term inference of population size and habitat use in a socially dynamic population of wild western lowland gorillas. Conservation Genetics. 2019;20(6):1303-14. doi: 10.1007/s10592-019-01209-w. PubMed PMID: WOS:000491084800008.

14. Seiler N, Robbins MM. Ecological correlates of space use patterns in wild western lowland gorillas. American Journal of Primatology. 2020;82(9). doi: 10.1002/ajp.23168. PubMed PMID: WOS:000544219600001.

15. Breuer T, Robbins AM, Olejniczak C, Parnell RJ, Stokes EJ, Robbins MM. Variance in the male reproductive success of western gorillas: acquiring females is just the beginning. Behavioral Ecology and Sociobiology. 2010;64(4):515-28. PubMed PMID: WOS:000274433600002.

16. Watts DP. Infanticide in mountain gorillas - new cases and a reconsideration of the evidence. Ethology. 1989;81(1):1-18. PubMed PMID: ISI:A1989T412400001.

17. Robbins MM. A demographic analysis of male life history and social structure of mountain gorillas. Behaviour. 1995;132:21-47. PubMed PMID: ISI:A1995QP20900002.

18. Stoinski TS, Vecellio V, Ngaboyamahina T, Ndagijimana F, Rosenbaum S, Fawcett KA. Proximate factors influencing dispersal decisions in male mountain gorillas, *Gorilla beringei beringei*. Animal Behaviour. 2009;77(5):1155-64. PubMed PMID: ISI:000265283900022.

19. Brncic T. Results of the 2016-2017 large mammal survey of teh Ndoki-Likouala landscape. Wildlife Conservation Society, 2019.

20. Breuer T, Hockemba MBN, Strindberg S. Factors Influencing Density and Distribution of Great Ape Nests in the Absence of Human Activities. International Journal of Primatology. 2021;42(4):640-65. doi: 10.1007/s10764-021-00229-z. PubMed PMID: WOS:000665686600001.

21. Fay JM, Carroll R, Peterhans JCK, Harris D. Leopard attack on and consumption of gorillas in the Central-African-Republic. Journal of Human Evolution. 1995;29(1):93-9. PubMed PMID: ISI:A1995RJ36600004.

22. Klailova M, Casanova C, Henschel P, Lee P, Rovero F, Todd A. Non-Human Predator Interactions with Wild Great Apes in Africa and the Use of Camera Traps to Study Their Dynamics. Folia Primatologica. 2012;83(3-6):312-28. doi: 10.1159/000342143. PubMed PMID: WOS:000314241200010.

23. Mavinga FB. A camera trap assessment of factors influencing leopard (*Panthera pardus*) habitat use in the Nouabalé-Ndoki National Park, Republic of Congo: University of Cape Town; 2018.

24. Parnell RJ. Group size and structure in western lowland gorillas (*Gorilla gorilla gorilla*) at Mbeli Bai, Republic of Congo. American Journal of Primatology. 2002;56(4):193-206. PubMed PMID: ISI:000174552200001.

25. Manguette ML, Robbins AM, Breuer T, Stokes EJ, Parnell RJ, Robbins MM. Intersexual conflict influences female reproductive success in a female-dispersing primate. Behavioral Ecology and Sociobiology. 2019;73(9). doi: 10.1007/s00265-019-2727-3. PubMed PMID: WOS:000485186000001.

26. Robbins AM, Robbins MM, Fawcett K. Maternal investment of the Virunga mountain gorillas. Ethology. 2007;113(3):235-45. PubMed PMID: ISI:000244274300004.

27. Manguette ML, Robbins AM, Breuer T, Stokes EJ, Parnell RJ, Robbins MM. Female dispersal patterns influenced by male tenure duration and group size in western lowland gorillas. Behavioral Ecology and Sociobiology. 2020;74(7). doi: 10.1007/s00265-020-02863-8. PubMed PMID: WOS:000540151600001.

28. Colchero F, Aburto JM, Archie EA, Boesch C, Breuer T, Campos FA, et al. The long lives of primates and the 'invariant rate of ageing' hypothesis. Nature Communications. 2021;12(1). doi: 10.1038/s41467-021-23894-3. PubMed PMID: WOS:000664863000009.
