# Supplementary material for: Population dynamics of western gorillas at Mbeli Bai
Source: PLoS One. 2022 Oct 19;17(10):e0275635. doi: 10.1371/journal.pone.0275635 (PMC9581538; doi:10.1371/journal.pone.0275635)
Supplement: Attachment — Submitted filename: REVIEWERS COMMENTS.docx [file pone.0275635.s003.docx]

**POPULATION DYNAMICS OF WESTERN GORILLAS AT MBELI BAI**

**REVIEWERS’ COMMENTS:**

This is a well-established study. No corrections for this article
