# Supplementary material for: Population dynamics of western gorillas at Mbeli Bai
Source: PLoS One. 2022 Oct 19;17(10):e0275635. doi: 10.1371/journal.pone.0275635 (PMC9581538; doi:10.1371/journal.pone.0275635)
Supplement: Attachment — Submitted filename: Response to Reviewers.docx [file pone.0275635.s004.docx]

**POPULATION DYNAMICS OF WESTERN GORILLAS AT MBELI BAI**

**REVIEWERS’ COMMENTS:**

# Reviewer #1:

The study is well comprehensive addressing density dependance and population dynamics of western gorillas at Mbeli Bai. I have no corrections for this research paper. The author investigates the population dynamics of western gorillas in Mbeli Bai, a swampy forest clearing within the Nouabalé-Ndoki National Park (NNNP) in the Republic of Congo, over a 25-year period. Despite their widespread range in Central Africa, western gorillas (Gorilla gorilla gorilla) are critically endangered. The entire population of western gorillas is estimated to be 330,000, with a 2.7 percent annual decline owing to illicit murders, sickness, and habitat destruction.

*Thank you for the comments.*

# Reviewer #2:

The abstract needs some minor corrections.
*We have now reworded some sentences in the Abstract.*

The introduction is too clumsy, losing clarity and relevance. Some of the observations are out of context and need to be streamlined. The objective of the study was not mentioned properly.
*To streamline the Introduction, we removed most of the fifth paragraph which included methods that are not relevant to this study. On line 73 of the original pdf, we reworded the sentence to explicitly state the overall objective of the study. More detailed objectives are provided in the last paragraph of the Introduction (e.g.,“to determine whether Mbeli is a source, a sink, or a refuge”, “to look for potential effects of density dependence upon female reproductive success”, etc.).*

Methods require additional clarification on the growth rate in equation number 3. The authors also missed important variables like ecological and environmental parameters and their impact on the population. Is there any correlation between the emergence of bais and stable population growth?
*We have now added an example to illustrate the conversion from a monthly growth rate to an annual growth rate using Equation 3. We agree that it would be worthwhile to include any temporal variations in ecological conditions around the bai, but unfortunately such information was not available. We are unaware of any correlation between the emergence of bais and population growth.*

I am not clear about the percentile figure of gorillas presented in the first paragraph of the result. Is this figure inclusive of perinatal mortality, disease-related deaths, infanticides, and so on?
*After line 180 of the original pdf, we have now stated that the assumed deaths could be due to predation, disease, infanticides, or other causes. The study was unlikely to detect perinatal mortality because the gorillas were not observed daily, and it is not visually apparent when they are pregnant.*

 Line no 409-411:  Is there any evidence of lower population growth or losses prior to the establishment of the Nouabalé-Ndoki National Park (NNNP) in 1993?
*We are unaware of any studies of population changes before 1993.*

Lines 434-462: The observations are intriguing, and one inference line should be drawn from them at the end of the second paragraph.

*On line 462, we have now stated that density dependence of male mating competition could have a greater impact on the population dynamics of the Virunga mountain gorillas than other species.*

# Editor

1. Please ensure that your manuscript meets PLOS ONE's style requirements, including those for file naming. The PLOS ONE style templates can be found at 
<https://journals.plos.org/plosone/s/file?id=wjVg/PLOSOne_formatting_sample_main_body.pdf> and 
<https://journals.plos.org/plosone/s/file?id=ba62/PLOSOne_formatting_sample_title_authors_affiliations.pdf>

*Sorry, but we couldn’t see how our manuscript differs from the style requirements in those links. Could you please be more specific about any changes that are needed?*

2. In your Data Availability statement, you have not specified where the minimal data set underlying the results described in your manuscript can be found. PLOS defines a study's minimal data set as the underlying data used to reach the conclusions drawn in the manuscript and any additional data required to replicate the reported study findings in their entirety. All PLOS journals require that the minimal data set be made fully available. For more information about our data policy, please see <http://journals.plos.org/plosone/s/data-availability>.

*We revised the Data Availability statement to indicate that the data is in a Supporting Information file. We named the file “ArchiveData_PopulationDynamics_WesternGorillas”, but the name somehow got encrypted when we uploaded it, which might make it harder to find.*
